# Accelerated fibrosis and apoptosis with ageing and in atrial fibrillation: Adaptive responses with maladaptive consequences

**DOI:** 10.3892/etm.2013.899

**Published:** 2013-01-16

**Authors:** GUO-JUN XU, TIAN-YI GAN, BAO-PENG TANG, ZU-HENG CHEN, AILIMAN MAHEMUTI, TAO JIANG, JIAN-GUO SONG, XIA GUO, YAO-DONG LI, HAI-JUN MIAO, XIAN-HUI ZHOU, YU ZHANG, JIN-XIN LI

**Affiliations:** 1 Department of Cardiology, First Affiliated Hospital, Xinjiang Medical University, Urumqi, Xinjiang 830011;; 2 Department of Animal Experiment, First Affiliated Hospital, Xinjiang Medical University, Urumqi, Xinjiang 830011;; 3 Department of Electrophysiology, First Affiliated Hospital, Xinjiang Medical University, Urumqi, Xinjiang 830011;; 4 Department of Molecular Biology, Xinjiang Medical University, Urumqi, Xinjiang 830054, P.R. China

**Keywords:** atrial fibrillation, ageing, structural remodeling, atrial fibrosis, apoptosis

## Abstract

The aim of this study was to investigate whether abnormal expression of matrix metalloproteinase (MMP)-9/tissue inhibitors of MMPs (TIMP)-1 and B cell lymphoma 2 (BCL-2)/BCL-2-associated X protein (BAX) are correlated with the characteristic accelerated fibrosis and apoptosis during ageing and in atrial fibrillation (AF). Four groups of dogs were studied: adult dogs in sinus rhythm (SR), aged dogs in SR, adult dogs with AF induced by rapid atrial pacing and aged dogs with AF induced by rapid atrial pacing. The mRNA and protein expression levels of the target gene in the left atrium were measured by quantitative reverse transcription-polymerase chain reaction (RT-PCR) and western blot analysis. Pathohistological and ultrastructural changes were assessed by light and electron microscopy. The apoptotic indices of myocytes were detected by terminal deoxynucleotidyl transferase-mediated deoxyuridine triphosphate (dUTP) nick end labeling (TUNEL). The mRNA and protein expression levels of MMP-9 and BAX and those of TIMP-1 and BCL-2 were significantly upregulated and down-regulated, respectively, in the aged groups compared with the adult groups. Compared with the control groups, the adult and aged groups with AF exhibited significantly increased mRNA and protein expression levels of MMP-9 and BAX and decreased expression levels of TIMP-1 and BCL-2. Samples of atrial tissue demonstrated abnormal pathohistological and ultrastructural changes, accelerated fibrosis and apoptosis. MMP-9/TIMP-1 and BCL-2/BAX hold potential for use as substrates conducive to AF and their abnormal expression plays a major role in structural remodeling of the atrium.

## Introduction

Atrial fibrillation (AF) is the most common cardiac arrhythmia and affects millions of individuals worldwide. The incidence of AF increases with age (1,2). Although the details are poorly understood, the persistence of AF is considered a result of atrial remodeling (3). Atrial remodeling includes electrical, structural and contractile remodeling processes, which are the primary contributors to the development and maintenance of AF (3,4). Structural remodeling may be an adaptive process (dedifferentiation of cardiomyocytes) aimed at protecting the atrial myocytes or a maladaptive process (degeneration of cells with fibrotic replacement) (4,5). Despite the different characteristics, all adaptive processes are ultimately processes of programmed cell survival (6). Certain myocytes with structural alterations are able to activate a programmed cell death pathway (7). Moreover, these dynamic processes may be modulated by the expression of B cell lymphoma 2 (BCL-2; which protects cells from apoptosis) and BCL-2-associated X protein (BAX; which opposes the effects of BCL-2, thereby promoting apoptosis) (8).

Previous studies have indicated that the homogeneous conduction of atrial activity not only relies on cardiomyocyte integration but is also affected by the extracellular matrix (ECM) among the atrial myocytes (9,10). Atrial fibrosis (abnormal deposition of ECM proteins in the atrium) may be associated with the substrates of AF by increasing the heterogeneity of atrial conduction and playing a significant role in the maintenance of this type of arrhythmia (11,12). Interstitial fibrosis may facilitate local intra-atrial conduction block and increase atrial susceptibility to AF, as well as form stable local sources for atrial micro-reentry and AF induction (13,14). In addition, matrix metalloproteinases (MMPs) and their endogenous inhibitors, tissue inhibitors of MMPs (TIMPs), are regarded as potential etiological agents in atrial fibrotic remodeling (15).

However, the mechanisms of atrial fibrosis during ageing and/or in AF remain unclear. The purpose of this study was to investigate whether abnormal expression of MMP-9/TIMP-1 and BCL-2/BAX are correlated with histological and ultra-structural changes, including the characteristic accelerated fibrosis and apoptosis during ageing and/or in AF induced by chronic rapid atrial pacing. We also sought to identify potential substrates for AF.

## Materials and methods

### Animal preparation

Fourteen adult (1–3 year old) and 14 aged (>8 year old) mongrels weighing 18–26 kg were obtained from the Animal Center of Xinjiang Medical University (Urumqi, China). The ages of the dogs were estimated by a veterinarian based on standard measurements for age, including dentition, coat and eye condition, as well as musculoskeletal and conformational descriptors. The dogs were kept in temperature-controlled housing under a 12/12 h light/dark cycle and fed a standard laboratory diet and water *ad libitum*. The Animal Care and Use Committee of the Xinjiang Medical University approved all experiments in accordance with the National Institutes of Health Guide for the Care and Use of Laboratory Animals (Publication No. 85–23, revised 1985).

### Experimental design

Six-lead electrocardiogram (ECG) measurements were made on conscious dogs resting quietly to confirm sinus rhythm (SR). Echocardiograms were performed to exclude structural heart disease. The animals were randomly divided into four groups of seven: the adult SR, aged SR, adult AF and aged AF groups. AF was induced by chronic rapid atrial pacing and defined as the persistence of AF for ≥5 days.

### Induction of AF

Animals were anesthetized with pentobarbital sodium (30 mg/kg i.v.) and ventilated with 1.5–2% isoflurane and 2 l/min O_2_. In total, 0.15 mg/kg morphine sulfate was injected into the epidural space for postoperative analgesia. Under sterile conditions, a right intercostal thoracotomy was performed, the pericardium was opened and the heart was suspended in a pericardial cradle. A lead was attached to the epicardium of the left atrial (LA) appendage. It was tunneled subcutaneously and connected to a pulse generator (Department of Electronic Engineering, Fudan University, Shanghai, China). Pulse generators were implanted in subcutaneous pockets on the left posterior chest wall. Once the incisions were closed and the dogs had recovered from the anesthesia, they were monitored for 2–3 days in the recovery room before being moved for routine care. They were treated prophylactically with cefazolin (25 mg/kg i.v. BID) for 2 days after surgery. They were allowed to stabilize for 1 week and were then subjected to LA appendage pacing at 600 bpm to induce persistent AF. Dogs that exhibited persistent AF for ≥5 days were analysed *in vitro*.

### Morphological evaluation

Tissues from the LA wall were immediately fixed in 4% paraformaldehyde at 4°C and embedded in paraffin. Light microscopy was performed using semi-thin sections (2 *μ*m) stained with hematoxylin-eosin and Masson’s trichrome stains. At least two sections per atrial site were examined and ≥200 cells per section were analyzed to quantify the extent of myolysis in the cardiomyocytes. The extent of cell change was evaluated only in cells in which the nucleus was present in the plane of the section. The myolytic area of the cardiomyocytes was measured with a digital imaging system (Motic Images Advanced, Richmond, BC, Canada). Cells were scored as mildly myolytic if myolysis involved 10–25% of the cytosol and as severely myolytic if >25% of the sarcomeres were absent.

For electron microscopy, ultrathin sections (50–100 nm) were cut from each sample, counterstained with uranium acetate and lead citrate and then examined under a transmission electron microscope at high magnification (×10,000) (Philips 201; Philips, Sunnyvale, CA, USA) by two professional staff.

### Terminal deoxynucleotidyl transferase-mediated deoxyuridine triphosphate (dUTP) nick end labeling (TUNEL) assay

The sections were transferred to xylene and rehydrated in decreasing concentrations of alcohol. Slides were then incubated (10 min, room temperature) with 10 *μ*g proteinase K (Sigma-Aldrich, St. Louis, MO, USA) per 1 ml phosphate-buffered saline (PBS). Endogenous peroxidase (POD) was inactivated with an ImmunoPure POD suppressor (Pierce Biotechnology Inc., Rockford, IL, USA) for 30 min. Tissue sections permeabilized with 1% Triton X-100 (4°C, 2 min) were stained with an *in situ* cell detection POD system (Boehringer Mannheim, Mannheim, Germany). DNA strand breaks were identified by labeling free 3′-OH termini with dUTP-fluorescein isothiocyanate (FITC) using TUNEL. Incorporated fluorescein was detected with an anti-fluorescein antibody Fab fragment from sheep conjugated with horse-radish POD. Following the reaction with metal-enhanced diaminobenzidine (DAB) (Boehringer Mannheim), sections were counterstained with hematoxylin. Positive controls consisted of fixed and permeabilized sections incubated with DNase I (1 *μ*g/ml; 10 min, room temperature). For negative controls, sections were incubated in labeling solution without terminal deoxynucleotidyl transferase. The percentage of TUNEL-positive nuclei was calculated by examining 50 randomly selected fields per section corresponding to ∼700 cells at high magnification (×400).

### Detection of gene expression

Total RNA was extracted from samples of the LA wall using TRIzol (Invitrogen Life Technologies, Carlsbad, CA, USA). The expression levels of target genes were measured by real-time quantitative reverse transcription-polymerase chain reaction (RT-PCR) using SYBR-Green qPCR master mix (Bio-Rad, Hercules, CA, USA) in a 20 *μ*l reaction volume containing 50 ng cDNA. All reactions were performed in triplicate and included a negative control. PCR was carried out using an ABI Prism 7500 Sequence Detection System (Applied Biosystems, Carlsbad, CA, USA) under the following conditions: 2 min at 50°C, 10 min at 95°C and 40 cycles of 15 sec at 95°C and 1 min at 60°C. Relative quantification of mRNA levels was obtained using the 7500 system, which performs comparative analysis. Fluorescence signals were normalized to the housekeeping gene, β-actin. The comparative threshold cycle (CT) relative quantification method was used (ΔΔCT). For every sample, each gene was quantified in duplicate in three separate experiments. The values were averaged and then used to calculate 2^−ΔΔCT^, which corresponds to the expression relative to β-actin. The amplicons of expected size were confirmed by gel electrophoresis. The sequences of the genes studied were obtained from GenBank and the primers were designed using Primer 5.0 (Applied Biosystems). The amplicon size of the primer sequence and annealing temperature of the genes are presented in Table I.

### Assessment of protein expression

Protein was extracted from tissue samples of the LA wall with 5 mmol/l Tris-HCl (pH 7.4), 2 mmol/l ethylenediamine tetraacetic acid (EDTA), 5 *μ*g/ml leupeptin, 10 *μ*g/ml benzamidine and 5 *μ*g/ml soybean trypsin inhibitor, followed by tissue homogenization. All procedures were performed at 4°C. Equal amounts (100 *μ*g/sample) of LA membrane proteins were separated on 8% sodium dodecyl sulfate-polyacrylamide gel electrophoresis (SDS-PAGE) gels and transferred to polyvinylidene difluoride membranes. Membranes were blocked in 5% non-fat dry milk in TTBS (50 mmol/l Tris-HCl; 500 mmol/l NaCl, pH 7.5; 0.05% Tween-20) for 2 h (room temperature) and then incubated with the primary antibody (1:500 dilution) in 5% non-fat dry milk in TTBS for 4 h at room temperature. Membranes were then incubated with rabbit polyclonal anti-MMP-9 (Santa Cruz Biotechnology Inc., Santa Cruz, CA, USA) as well as rabbit polyclonal anti-TIMP-1, BCL-2 and BAX (Abcam, Cambridge, MA, USA). Membranes were washed three times in TTBS, re-blocked in 5% non-fat dry milk in TTBS (15 min) and then incubated with horseradish POD-conjugated goat anti-rabbit (1:5000) in 5% non-fat dry milk in TTBS (40 min). Immunoreactive bands were detected by Immun-Star horse-radish peroxidase (HRP) substrate (Bio-Rad) and quantified by densitometry analysis using Image Quant 350 and Image Quant TL-1 (GE Healthcare, Fairfield, CT, USA). Anti-β-actin antibodies (Santa Cruz Biotechnology) were used to control for equal protein loading and to normalize the target protein band intensity. All western blotting target bands were expressed quantitatively by normalization to the control band on the same lane. Western blotting band intensities were expressed as optical density (OD) units corresponding to densitometric band intensity following background subtraction divided by β-actin signal intensity for the same sample.

### Statistical analysis

SPSS 15.0 (SPSS Inc., Chicago, IL, USA) was used in the statistical analysis. Quantitative data are expressed as mean ± standard deviation (SD). Comparisons between the quantitative data were made using the t-test, whereas those for qualitative data were assessed with χ^2^ analysis. P<0.05 was considered to indicate a statistically significant difference.

## Results

### ECG data

ECG data for the adult and aged groups studied in SR are shown in Table II. The ECGs of the aged dogs revealed longer P-wave durations and P-wave dispersion compared with those of the adult dogs. These groups did not differ in other variables and the difference between these two groups in time to onset of persistent AF was not significant, with the adult dogs having developed persistent AF after 40±5 days and the aged dogs acquiring it after 52±7 days of atrial pacing (P>0.05).

### Fibrotic changes

**Figure 1.**
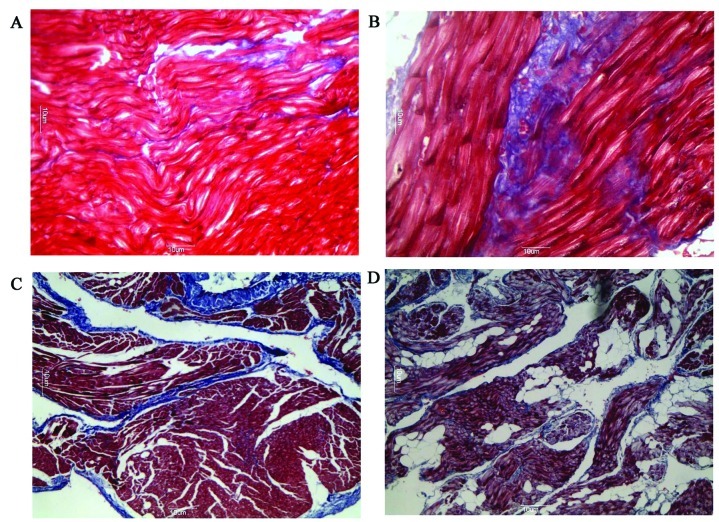
Representative sections of the LA free wall from the four groups of dogs showing myocytes in red and collagen in blue. The circular, clear spaces are interstitial fat. (A) Adult group; (B) aged group; (C) adult AF group; (D) aged AF group. Masson’s trichrome stain; original magnification, ×160. LA, left atrium; AF, atrial fibrillation.

As shown in Fig. 1, compared with those in the adult group, the myocardial fibres of tissue from the aged group appeared to be more compact and were closer together. Muscle bundles were separated by large strands of connective tissue and a significantly higher deposition of connective tissue was observed (4.1±0.9 vs. 8.4±1.0%; n=15; P<0.05). Additionally, the degree of myocardial fiber disarray was higher in the adult (4.1±0.9 vs. 14.7±2.1%; n=15; P<0.01) and aged (8.4±1.0 vs. 18.2±2.4%; n=15; P<0.01) groups with AF than in the corresponding groups in SR. The degree of myocardial fiber disarray among muscle bundles of tissue was greater in the aged group with AF than in the adult group with AF (14.7±2.1 vs. 18.2±2.4%; n=15; P<0.05).

### Cell ultrastructural changes

**Figure 2.**
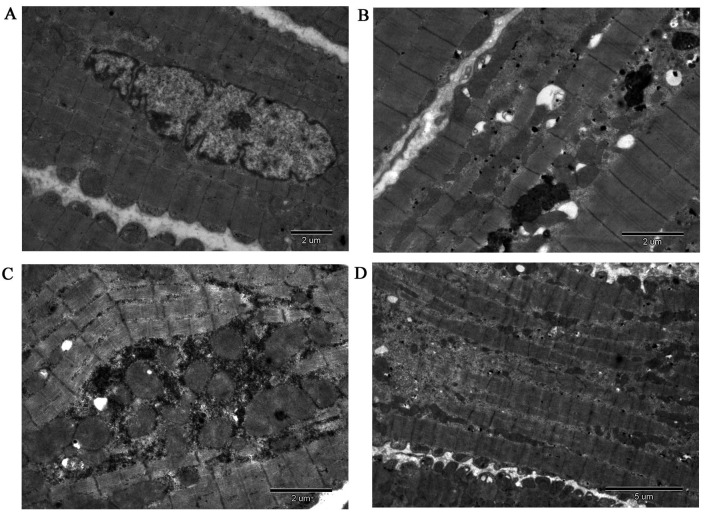
Typical examples of ultrastructural changes in atrial myocytes of the LA free wall from the four study groups. (A) Adult dogs; (B) aged dogs; (C) adult AF dogs; (D) aged AF dogs. Electron microscopy; original magnification, ×10,000. LA, left atrium; AF, atrial fibrillation.

The ultrastructure of the atrial myocardium was examined by electron microscopy and representative transmission electron micrographs are shown in Fig. 2. Atrial myocytes of the LA wall obtained from the adult dogs had a regular sarcomere organization, uniformly sized mitochondria and nuclei demonstrating normal clumping of chromatin at the nuclear membrane (n=3, samples from randomly selected adult dogs; Fig. 2A). Atrial myocytes of the LA wall obtained from the aged dogs presented an abnormal ultrastructure, with mild and severe sarcomere degeneration, mitochondrial swelling with a reduction in the density and organization of the cristae, karyopyknosis with chromatin margination to the nuclear membrane, expanding endoplasmic reticulum, increased glucogen and mildly compact and close myocardial fibers (n=3, samples from randomly selected aged dogs; Fig. 2B). Atrial myocytes of the LA wall obtained from the adult dogs with AF appeared to have a more abnormal ultrastructure, with severe sarcomere degeneration, more mitochondrial swelling, karyopyknosis indicating some cell apoptosis, several secondary lysosomes, expanding endoplasmic reticulum, decreased glucogen and irregular and disorderly myocardial fibers (n=3, samples from randomly selected adult dogs with AF; Fig. 2C). Atrial myocytes of the LA wall obtained from the aged dogs with AF also exhibited an abnormal ultrastructure, with more severe sarcomere degeneration, mitochondria showing vacuoles, more karyopyknosis indicating cell apoptosis, expanding endoplasmic reticulum and secondary lysosomes, as well as some disintegration of myofilaments (n=3, samples from randomly selected aged dogs with AF; Fig. 2D).

### Apoptotic indices

**Figure 3.**
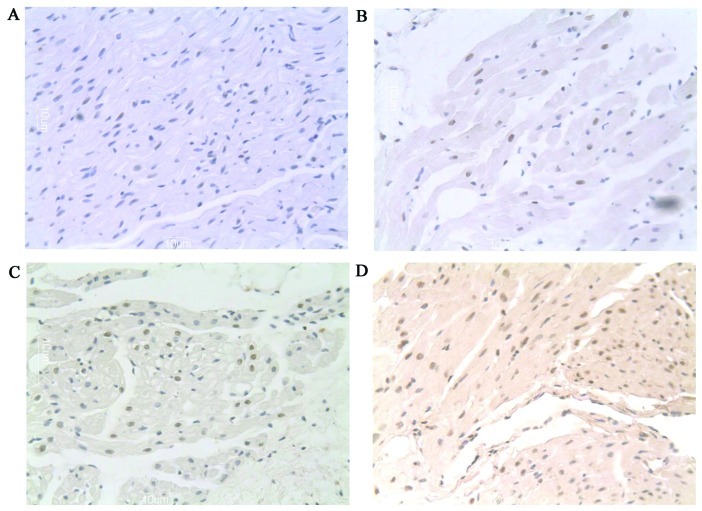
Staining of tissue sections from the four study groups with TUNEL. (A) Adult group; (B) aged group; (C) adult AF group; (D) aged AF group. Original magnification, ×400. TUNEL, terminal deoxynucleotidyl transferase-mediated deoxyuridine triphosphate nick end labeling; AF, atrial fibrillation.

As shown in Fig. 3, a higher percentage of myocytes with myolysis in aged dogs had TUNEL-positive nuclei compared with the adult group (22.0±5.45 vs. 32.9±3.4%; n=12; P<0.05). The majority of the nuclei with weak TUNEL staining were large with a uniform distribution of heterochromatin (Fig. 3A and B). Additionally, the frequency of apoptosis was significantly increased in the adult (22.0±5.45 vs. 33.4±3.9%; n=12; P<0.01) and aged (32.9±3.4 vs. 51.2±3.4%; n=12; P<0.001) groups with AF compared with their counterpart groups in SR, with the frequency in the aged group being higher. In addition, several nuclei decreased in size and were stained strongly by TUNEL, indicating that more extensive DNA cleavage was associated with these nuclear alterations (Fig. 3C and D).

### LA mRNA and protein expression of MMP-9/TIMP-1 and BCL-2/BAX

**Figure 4.**
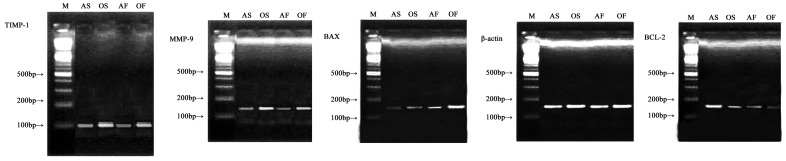
Representative gels of MMP-9/TIMP-1 and BCL-2/BAX mRNA expression in the LA myocardium. M, DL-2000 marker; AS, adult SR group; OS, aged SR group; AF, adult AF group; OF, aged AF group. MMP, matrix metalloproteinase; TIMP, tissue inhibitors of MMPs; BCL-2, B cell lymphoma 2; BAX, BCL-2-associated X protein; LA, left atrium; SR, sinus rhythm; AF, atrial fibrillation.

**Figure 5.**
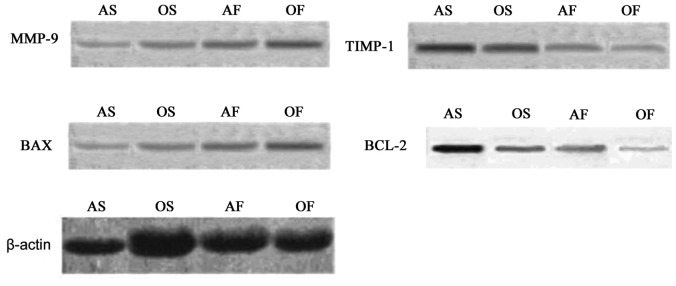
Representative immunoblots (western blotting) showing MMP-9/TIMP-1 and BCL-2/BAX protein expression in the LA myocardium. AS, adult SR group; OS, aged SR group; AF, adult AF group; OF, aged AF group; MMP, matrix metalloproteinase; TIMP, tissue inhibitors of MMPs; BCL, B cell lymphoma; BAX, BCL-2-associated X protein; LA, left atrium; SR, sinus rhythm; AF, atrial fibrillation.

As shown in Tables III and IV, and Figs. 4 and 5, the mRNA and protein expression levels of MMP-9 and BAX significantly increased (P<0.05), whereas the mRNA and protein expression of TIMP-1 and BCL-2 were down-regulated in the aged group compared with the adult group (P<0.05). Compared with the control groups, the adult and aged groups with AF exhibited significantly increased mRNA and protein expression levels of MMP-9 and BAX (P<0.05), with the expression of BAX being higher in the two AF groups (P<0.05). By contrast, the mRNA and protein expression levels of TIMP-1 and BCL-2 were down-regulated in the adult and aged groups with AF (P<0.05), with the expression of BCL-2 being lower in the two AF groups (P<0.05).

The protein expression levels of MMP-9 (r=0.348; P=0.019) and TIMP-1 (r=−0.331; P=0.027) were correlated with the degree of myocardial fiber disarray, whereas the protein expression levels of BAX (r= 0.451; P= 0.002) and BCL-2 were correlated with the frequency of apoptosis (r=−0.309; P= 0.038).

## Discussion

Myocardial fibrosis is a dynamic process during which normal collagen chains are degraded and replaced by fibrous interstitial deposits (9,10,13). MMPs are involved in matrix degradation and collagen synthesis. They play a crucial role in ECM homeostasis in a number of physiological and pathological situations, including during ageing and/or in AF (3,12,13,16). These proteolytic enzymes are regulated at transcriptional and translational levels, as well as by endogenous physiological inhibitors, including TIMPs (11,15). In the majority of disease processes, a dynamic interplay between these MMPs and the TIMPs (mainly MMP-9/TIMP-1) determines the phenotypic changes (14,17). Additionally, MMP-9 and TIMP-1 interact with tissue necrosis factors, angiotensin and other cytokines in the LA remodeling process in AF (17,18). Profibrotic signals act on the balance between MMPs and TIMPs (9,11,18). Thus, TIMP-1 deficiency has been shown to result in LA and left ventricular dilatation, cardiomyocyte hypertrophy and contractile dysfunction (10,15,17). The samples from our dog models of ageing and/or AF demonstrated impaired balance between MMP-9 and TIMP-1, with increased levels of MMP-9 and lack of TIMP-1 upregulation. In addition, our study demonstrated that the protein expression levels of MMP-9 and TIMP-1 were correlated with the levels of myocardial fibrosis in the LA with ageing and/or in AF. These results suggest that the altered MMP-9/TIMP-1 stoichiometry may result in loss of control of MMP-9 activity and contribute to the development of interstitial fibrosis in atrial remodeling.

Structurally, the most important change in aged atria is an enhancement of the fibrous tissue that is interspersed between myocytes. Additionally, these alterations predominate in fibrillating and ageing atria. Cardiac fibrosis is characterized by the excessive accumulation of fibrillar collagen in the extracellular space. Fibrosis is ubiquitous in the atria of the ageing heart (18). Interstitial fibrosis reduces electrical coupling in the heart (19) and significantly increases the complexity of the myocardial architecture by electrically insulating cardiac cells and/or muscle bundles (20,21), a direct consequence of which is that the typical uniform anisotropic conduction in the atrial myocardium is replaced by non-uniform anisotropic conduction (22,23). Studies have shown that such fibrosis preferentially affects lateral (or transverse) connections over longitudinal cell-cell connections (24,25), resulting in a much slower and zigzag transverse propagation (26,27). Thus, a premature response occurring in the aged atrial myocardium has a higher probability of undergoing a unidirectional block from an imbalance between source/sink currents and initiating reentry due to the underlying arrhythmogenic substrate created as a result of the electrical and/or structural remodeling (14,27,28).

The results of the current study indicate that ageing and/or fibrillating atria contain a number of apoptotic myocytes and that myocytes with myolysis may exhibit several features of dedifferentiation. We identified an impaired balance between BAX and BCL-2, with increased levels of BAX and lower levels of BCL-2 with ageing and/or in AF and determined that the protein expression of BAX and BCL-2 were correlated with the frequency of apoptosis in LA organisation with ageing and/or in AF. A balance therefore exists between protective anti-apoptotic signals (which likely take part in the molecular basis of the ‘cell survival syndromes’ described above) and pro-apoptotic pathways. Chronic or repetitive stress with ageing and/or in AF may lead to the progressive downregulation of protective mechanisms and sustained activation of pathways that eventually induce apoptosis.

Several observations relate the onset of apoptosis to metabolic dysfunction (6,7,8,29). To begin with, a major determinant of the apoptosis cascade is mitochondrial destabilization, characterized by loss of membrane potential, generation of reactive oxygen species and liberation of cytochrome *c*(6,7,8,30). As mitochondria are the main site of cardiac adenosine triphosphate (ATP) production, it is not surprising that apoptosis is related to ATP depletion (6,7,8,31). Decreased ATP content promotes the transfer of the pro-apoptotic protein BAX to the mitochondria (32). Insertion of BAX into the mitochondrial membrane creates pores through which cytochrome *c* is extruded to the cytosol (33). In other cell types, activation of the apoptotic pathway is accompanied by an impairment of glucose uptake and downregulation of the anerobic production of ATP (31,32,34). Interestingly, overexpression of BCL-2 counteracts the onset of apoptosis under conditions of ATP depletion (30,33,35). This protective effect is multifactorial, including an inhibition of BAX-induced release of cytochrome *c* and inhibition of APAF-1 (30,31,35). BCL-2 is now well established as an anti-apoptotic protector in cardiac cells. Studies have suggested that BCL-2 induces a state of ‘metabolic hibernation’ that improves cell resistance in stress conditions (35,36). As apoptosis requires energy, severe depletion of ATP is followed by necrosis rather than apoptosis (37,38). Cardiac apoptosis therefore results from ‘mild’ but repetitive or prolonged episodes of stress (ischemia, stretch or overload with ageing and/or in AF), which progressively downregulates protective mechanisms (downregulation of BCL-2 expression) and activates pro-apoptotic pathways (overexpression of BAX). When adaptive mechanisms of cardiac survival are no longer able to sustain cellular homeostasis, the disturbance of energy metabolism, contractile function and gene expression trigger cardiac apoptosis.

We did not subject the animals in our models of chronic AF to atrioventricular nodal ablation, rendering excessively rapid ventricular rates accompanied by rapid atrial pacing inevitable. In fact, the majority of the dogs with chronic AF in this study had a certain degree of atrial dilation, suggesting chronic hemodynamic overload of their atria besides AF, which may be an important factor in triggering a programmed cell death pathway and atrial fibrosis. For instance, abnormal levels of resting tension have been shown to induce apoptosis in ventricular myocytes (29–31). This mechanism may be particularly important for myocytes of thin atrial walls, in which even moderate hemodynamic overload may induce substantial overstretching. This may also explain why atrial samples from the aged dogs with AF, which often had a greater degree of hemodynamic overload of the atria, also had marked histological abnormalities and apoptotic cells.

## Figures and Tables

**Table I.** Primer sequence and amplicon size of genes.

Gene	Primer sequence	Amplicon size (bp)	Annealing temperature (°C)
β-actin	F: 5′-AAGGACCTGTATGCCAACACA-3′		
	R: 5′-ATCCACACAGAATACTTGCGTT-3′	152	57
MMP-9	F: 5′-GACGCTATGGGCTATGAGTTAC-3′		
	R: 5′-AGTCCAGGTAGCCCTTTAGGT3′	165	58
TIMP-1	F: 5′-TGGATTACAATGAGGCGAAG-3′		
	R: 5′-AGACCCTTCAATTCGGTATCA-3′	112	60
BCL-2	F: 5′-CACAAGAGCCAAGGCTACCT-3′		
	R: 5′-CAGGAAAGCAGGAAGTCTCAA-3′	158	58
BAX	F: 5′-ATTGAGAAACGATTTGCCTACA-3′		
	R: 5′-GGGAAATGGCTTATTCTCCTTTGCTT-3′	187	59

MMP, matrix metalloproteinase; TIMP, tissue inhibitors of MMPs; BCL-2, B cell lymphoma 2; BAX, BCL-2-associated X protein.

**Table II.** ECG data of adult and aged canines in SR.

Group	P (msec)	PWD (msec)	PR (msec)	QRS (msec)	QT (msec)
Adult	66.1±6.4	19.1±4.1	123.9±7.2	63.1±4.3	248.9±11.7
Aged	75.9±5.3^a^	26.7±3.1^a^	130.0±7.7	64.7±5.4	246.5±17.3

Data are presented as mean ± standard error of the mean (n=7 for adult and aged groups).

a P<0.05 vs. the adult group. ECG, electrocardiography; PWD, P-wave dispersion; SR, sinus rhythm.

**Table III.** Comparison of target gene mRNA levels between the atrial fibrillation groups and the sinus rhythm groups.

Group	n	MMP-9	TIMP-1	BAX	BCL-2
SR adult	7	1.1483±0.2371	3.1602±0.3029	1.2520±0.2831	2.9571±0.3745
SR aged	7	1.8520±0.3029^a^	2.1139±0.2273^a^	1.9520±0.1824^a^	1.8623±0.3382^a^
AF adult	7	2.1372±0.2981^a^	1.9126±0.3812^a^	2.7361±0.2937^a^	1.5620±0.2490^a^
AF aged	7	2.8072±0.3369^b^	1.1683±0.1927^b^	3.2532±0.3271^b^	0.8524±0.2176^b^

Data are presented as mean ± standard deviation.

a P<0.05, compared with the adult SR group;

b P<0.05, compared with the aged SR group. MMP, matrix metalloproteinase; TIMP, tissue inhibitors of MMPs; BCL-2, B cell lymphoma 2; BAX, BCL-2-associated X protein; SR, sinus rhythm; AF, atrial fibrillation.

**Table IV.** Comparison of target protein levels between the atrial fibrillation groups and the sinus rhythm groups.

Group	n	MMP-9	TIMP-1	BAX	BCL-2
SR adult	7	0.1738±0.0329	0.7620±0.0572	0.2165±0.0263	0.6850±0.0562
SR aged	7	0.3093±0.0462^a^	0.5833±0.0429^a^	0.4281±0.0390^a^	0.5192±0.0509^a^
AF adult	7	0.4473±0.0528^a^	0.3982±0.0320^a^	0.5018±0.0472^a^	0.3278±0.0297^a^
AF aged	7	0.6172±0.0627^b^	0.2207±0.0313^b^	0.7163±0.0572^b^	0.1629±0.0162^b^

Data are presented as mean ± standard deviation.

a P<0.05, compared with the adult SR group;

b P<0.05, compared with the aged SR group. MMP, matrix metalloproteinase; TIMP, tissue inhibitors of MMPs; BCL-2, B cell lymphoma 2; BAX, BCL-2-associated X protein; SR, sinus rhythm; AF, atrial fibrillation.
